# Noble Metal Nanoparticles for Biosensing Applications

**DOI:** 10.3390/s120201657

**Published:** 2012-02-07

**Authors:** Gonçalo Doria, João Conde, Bruno Veigas, Leticia Giestas, Carina Almeida, Maria Assunção, João Rosa, Pedro V. Baptista

**Affiliations:** 1 CIGMH, Departamento de Ciências da Vida, Faculdade de Ciências e Tecnologia, Universidade Nova de Lisboa, Campus de Caparica, 2829-516 Caparica, Portugal; E-Mails: doria_go@fct.unl.pt (G.D.); conde.bio@gmail.com (J.C.); bmrveigas@gmail.com (B.V.); l.giestas@fct.unl.pt (L.G.); msa.carina@gmail.com (C.A.); maria.sm.assuncao@gmail.com (M.A.); joao.rosa@fct.unl.pt (J.R.); 2 Instituto de Nanociencia de Aragón, Universidad de Zaragoza, Campus Riìo Ebro, Edifiìcio I+D, Mariano Esquillor, s/n, 50018 Zaragoza, Spain; 3 CENIMAT/I3N, Departamento de Ciência dos Materiais, Faculdade de Ciências e Tecnologia, Universidade Nova de Lisboa, Campus de Caparica, 2829-516 Caparica, Portugal; 4 REQUIMTE, Departamento de Química, Faculdade de Ciências e Tecnologia, Universidade Nova de Lisboa, Campus de Caparica, 2829-516 Caparica, Portugal

**Keywords:** nanotechnology, noble metal nanoparticles, biosensors, molecular diagnostics, immunoassays, DNA, RNA, nucleic acids, proteins, antibody

## Abstract

In the last decade the use of nanomaterials has been having a great impact in biosensing. In particular, the unique properties of noble metal nanoparticles have allowed for the development of new biosensing platforms with enhanced capabilities in the specific detection of bioanalytes. Noble metal nanoparticles show unique physicochemical properties (such as ease of functionalization via simple chemistry and high surface-to-volume ratios) that allied with their unique spectral and optical properties have prompted the development of a plethora of biosensing platforms. Additionally, they also provide an additional or enhanced layer of application for commonly used techniques, such as fluorescence, infrared and Raman spectroscopy. Herein we review the use of noble metal nanoparticles for biosensing strategies—from synthesis and functionalization to integration in molecular diagnostics platforms, with special focus on those that have made their way into the diagnostics laboratory.

## 1. Introduction

In the era of nanotechnology, noble metal nanoparticles (NPs) have played an important role in the development of new biosensors and/or in the enhancement of existing biosensing techniques to fulfill the demand for more specific and highly sensitive biomolecular diagnostics. The unique physicochemical properties of such metals at the nanoscale have led to the development of a wide variety of biosensors, such as: (i) nanobiosensors for point of care disease diagnosis, (ii) nanoprobes for *in vivo* sensing/imaging, cell tracking and monitoring disease pathogenesis or therapy monitoring and (iii) other nanotechnology-based tools that benefit scientific research on basic biology [1–5]. In fact, NPs are, in general, one of the most common nanotechnology-based approaches for developing biosensors, due to their simplicity, physiochemical malleability and high surface areas [6]. They can measure between 1 to 100 nm in diameter, have different shapes and can be composed of one or more inorganic compounds, such as noble metals, heavy metals, iron, *etc.* The majority of them exhibit size-related properties that differ significantly from those observed in microparticles or bulk materials. Depending on their size and composition we can observe peculiar properties, such as quantum confinement in semiconductor nanocrystals, surface plasmon resonance in some metal NPs and superparamagnetism in magnetic materials. Noble metal NPs, in particular gold and silver NPs, are among the most extensively studied nanomaterials and have led to the development of innumerous techniques and methods for molecular diagnostics, imaging, drug delivery and therapeutics. Most of their unique physicochemical properties at the nanoscale, such as Localized Surface Plasmon Resonance (LSPR), have been explored for the development of new biosensors. This review will focus on these unique physicochemical properties of noble metal NPs that have thus far been explored for the development of new highly sensitive and specific biosensing techniques, favoring those that have already been successfully tested with biological samples. While some recent reports have addressed specific bio-application for noble metal NPs, such as molecular diagnostics and therapy [5,7] or cancer applications [8], and others have focused on the bio-applications of a specific type of noble metal NP, mostly gold NPs [9], here we aim at presenting an overview on the general principals and up to date applications of all noble metal NPs used for the development of biosensors.

## 2. Noble Metal Nanoparticles for Biosensing

### 2.1. Synthesis and Functionalization of Noble Metal Nanoparticles

Numerous techniques have been developed to synthesize noble metal NPs, including chemical methods (e.g., chemical reduction, photochemical reduction, co-precipitation, thermal decomposition, hydrolysis, *etc.*) and physical methods (e.g., vapor deposition, laser ablation, grinding, *etc.*), whose ultimate goal is to obtain NPs with a good level of homogeneity and provide fine control over size, shape and surface properties, in order to better take advantage of their unique physicochemical properties for biosensing [10].

The majority of the biosensing applications based on NPs that have been developed thus far are based on gold NPs, due to their unique optical properties and ease of derivatization with different biomarkers in aqueous solution [11–13]. These gold NPs, also known as colloidal gold, can be easily synthesized in sizes ranging between 3 and 200 nm in diameter and in different shapes, being the most common the quasi-spherical shape, mainly due to their surface energy that favors the formation of spherical particles. Generally, the method of choice to synthesize quasi-spherical gold NPs is the chemical reduction of Au(III) to Au(0) ions using sodium citrate as a reducing agent, a method first developed by Turkevich [14] and latter optimized by Frens [15]. In this approach, the citrate acts both as reducing agent and as capping agent which, as the gold NPs form, prevents the NPs from forming larger particles and simultaneously conferring them a mild stability due to electrostatic repulsion between citrate-capped gold NPs [16]. Recent modifications of the Turkevich method have allowed a better distribution and control over the size of the gold NPs, where a range between 9–120 nm can be achieved just by varying the citrate/Au ratio [17–19]. Alternatively, many other aqueous- and organic-based methodologies have been developed for the controlled synthesis of different noble metal NPs, including spherical or non-spherical, pure, alloy or core/shell NPs of gold, silver, platinum, palladium and/or rhodium [20–22]. The development of new biosensing and therapeutic applications based on noble metal NPs has been pushing forward the chemistry for their functionalization with different moieties such as nucleic acids, antibodies, biocompatible polymers, enzymes and other proteins, in a quest for an increased biocompatibility and targeting specificity [10,23].

Table 1 summarizes the different approaches for the biofunctionalization of noble metal NPs, including the main pros and cons of each approach, which can be consulted in more detail in the excellent review made by Sperling *et al.* [10].

A range of highly sensitive biosensing methods for nucleic acids, proteins, antibodies, enzymes and other biological molecules have been developed by exploring different physicochemical properties of the noble metal NPs, such as LSPR, fluorescence enhancement/quenching, surface-enhanced Raman scattering (SERS), electrochemical activity, *etc*. Among these biosensing methods, the colorimetric approaches have been the most explored and, due to their simplicity and portability, are one the most promising for future diagnostic methods at point-of-care. For this reason also, most methods have been based on silver and gold NPs, since these present unique optical properties within the visible wavelength range and are easy to synthesize and functionalize, with gold NPs being the most explored of all noble metal NPs. Nonetheless, even though in much less extension than silver and gold NPs, other noble metal NPs, such as platinum NPs, have also been used in biosensing applications, mostly by exploring their unique electrochemical properties.

### 2.2. Localized Surface Plasmon Resonance

**Figure 1.**
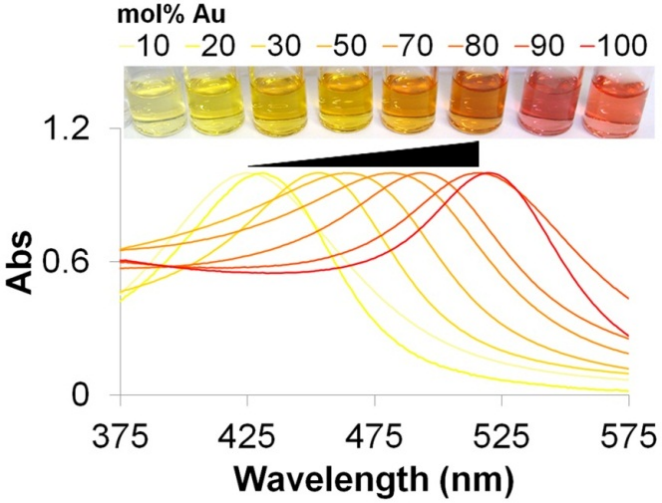
Example of LSPR modulation through different NP compositions. The LSPR absorption band of gold/silver alloy NPs increases to longer wavelengths with increasing amounts of gold.

One of the most explored characteristic of noble metal NPs for biosensing is their LSPR arising from the electromagnetic waves that propagate along the surface of the conductive metal [24]. When excited with an electromagnetic wave, such as light, most noble metal NPs produce an intense absorption and scattering due to the collective oscillation of the conduction electrons located at the NPs’ surface. In the particular case of gold and silver NPs, the LSPR yields exceptionally high absorption coefficients and scattering properties within the UV/visible wavelength range that allows them to have a higher sensitivity in optical detection methods than conventional organic dyes, making them the perfect candidates for colorimetric biosensing applications [25,26]. Moreover, their LSPR properties can be easily modulated according to their size, shape and composition [22,27]. Figure 1 illustrates the effect of nanoparticle composition in LSPR that has been already demonstrated beneficial for the development of new and highly sensitive biosensing methods [28,29].

Typically, colloidal solutions of spherical gold NPs (<40 nm) present a red color with their LSPR band centered at ca. 520 nm, while spherical silver NPs present a yellow color with their LSPR band centered at *ca.* 420 nm [25]. Both metals can also be combined in an alloy or core-shell conformation, presenting a LSPR band that can vary within the wavelength limits of pure metal NPs LSPR bands. In the case of the core-shell conformation, a dual LSPR peak characteristic of each pure metal can be observed, depending on the thickness of the metallic shell [27]. These LSPR bands are usually weakly dependent on the size of the NPs and the refractive index of the surrounding media, but strongly change with inter-particle distance, for example aggregation of NPs leads to a pronounced color change as a consequence of the plasmon coupling between NPs and a concomitant red-shift of the LSPR absorption band peak [30]. Most of the colorimetric biosensors based on gold and/or silver NPs have been developed considering these changes in color generated by the plasmon coupling between NPs upon aggregation, while other methods have used the LSPR properties of the noble metal NPs just as a colorful reporter (*i.e.*, making use of their superb scattering and/or absorbance properties). Some are based on the unspecific adsorption of biomolecules to non-functionalized noble metal NPs, while others are based on functionalized noble metal NPs for increased specificity.

In the case of the non-functionalized NPs, Li *et al.* took advantage of the differential propensity of ssDNA and dsDNA adsorption to gold NPs to develop a biosensor for DNA detection [31]. The free bases of ssDNA molecules interact electrostatically with the negatively charged surface of gold NPs harboring a citrate capping, which confers an increased stability to the NPs upon increasing ionic strength. On the other hand, dsDNA molecules adsorb much less to the NPs’ surface and do not provide stability to increasing ionic strength induced aggregation of the NPs. Based on these observations, Li and co-workers combined gold NPs with citrate capping with a polymerase chain reaction (PCR) procedure, using ssDNA probes complementary to the amplicon. Whenever the template of the PCR is amplified, the ssDNA probes hybridize with the amplicon and consequently become unavailable to adsorb to the gold NPs’ surface and confers them with increased stability to salt induced aggregation. This way, a positive result (*i.e.*, target amplification) yields a colorimetric change from red to blue upon salt addition, while in a negative result the color remains unchanged. This approach has also been successfully explored for the detection of SNPs [32,33], ultraviolet (UV)-induced mutagenic or carcinogenic DNA dimers [34], or to directly detect unamplified hepatitis C virus RNA isolated from clinical specimens [35]. Similarly, Xia and co-workers used the same approach to develop a nearly “universal” biosensor to detect a broad range of targets including nucleic acids, proteins, small molecules and inorganic ions, using conjugated polyelectrolytes and different ssDNA aptamers or probe molecules that mediated the target detection [36]. Ma *et al.* used gold nanorods in a CTAB solution to achieve a DNA target detection limit down to 0.1 pM [37] and Kanjanawarut *et al.* used peptide nucleic acids instead of ssDNA oligonucleotide probes to improve the differential aggregation between complementary and non-complementary targets [38]. Non-functionalized citrate-capped NPs have also been used to develop colorimetric biosensors for thiourea and melamine [39,40]. In these approaches, the presence of thiourea or melamine in the colloidal solution reduced the overall surface charges of silver or gold NPs, respectively, resulting in their aggregation and a colorimetric response correlating with the concentration of the targets (up to 0.8 nM for thiourea and 40 ppb for melamine).

Other approaches have used noble metal NPs functionalized with different biosensing molecules (e.g., ssDNA, antibodies, proteins or enzymes) in order to increase specificity of the methods. In 1996, Mirkin *et al.* described the use gold NPs functionalized with thiol-modified ssDNA probes (*i.e.*, gold nanoprobes) that would form a cross-linking network upon detection of a complementary ssDNA target by both gold nanoprobes [41]. This cross-linking network lead to the aggregation of the gold NPs causing a red-shift in the LSPR absorbance band to 574 nm. It was also possible to detect a single base mismatch through this cross-linking approach by controlling the temperature of denaturation of the cross-linked network, which presents a sharp melting transition [42]. This cross-linking approach has also been combined with a rolling circle amplification technique allowing the detection of single point mutations with 1 fM sensitivity [43]. Other methods for specific nucleic acid detection using gold NPs functionalized with ssDNA probes have been developed based on their differential non-cross-linking aggregation mediated by the increasing ionic strength of the solution [44–50]. In the case of the non-cross-linking method developed by Baptista and co-workers, the differential aggregation of the gold nanoprobes is evaluated upon salt addition to discriminate the presence of complementary, mismatched and non-complementary targets in solution [45–50]. Only the presence of a fully complementary target provides stability to the gold nanoprobes, while mismatched and non-complementary targets allow the nanoprobes to aggregate upon salt addition. This method has been thus far successfully applied to the detection of pathogenic agents [46,48] and single-nucleotide polymorphism (SNP)/single point mutations [50,28], as well as in gene expression analysis without the need for retro-transcription [47,49]. Moreover, the use of different gold/silver alloy nanoprobes in combination with the pure gold nanoprobes has allowed to create a multiplex DNA biosensor following this non-cross-linking approach [28,29]. Jian and co-workers also developed a non-cross-linking assay for the specific detection of a gene associated with sickle-cell anemia using fibrinogen-functionalized 56 nm gold NPs and a thrombin-binding aptamer assembled on gold NPs [51]. The detection was mediated through thrombin-induced aggregation of the fibrinogen-functionalized gold NPs allowing to achieve a limit of detection of 12 pM. The potency of the inhibition mediated by the thrombin-binding aptamer conjugated with gold NPs relative to thrombin also demonstrated to be highly dependent on the concentration of fully complementary DNA targets presenting a linear sensitivity in the concentration range of 20–500 pM.

Apart from the methods based on colorimetric alterations, other colorimetric methods have been developed using the noble metal NPs as a reporter. One example is the method for detecting nucleic acid sequences through the hybridization with gold nanoprobes in a chromatographic strip, also known as dipstick, as described by Kalogianni *et al.* [52]. In this approach, similarly to current pregnancy tests, biotinylated PCR products are hybridized to a specific oligo(dA)-tailed probe and loaded on the chromatographic stripe. As the buffer migrates through the stripe, the biotinylated PCR products are immobilized by streptavidin spotted in a specific location of the stripe. Finally, to detect the presence of the DNA target in the streptavidin spot, poly-dT gold nanoprobes are used to hybridize with the poly-dA probe. Other variants of this technique have also been described by Kalogianni *et al.* to fit a specific application (e.g., SNP detection, multiplex analysis) [53–55] and by Fan *et al.* to monitor protein-protein interactions using silver NPs instead of gold NPs [56]. Using a high-throughput microarray approach, Mirkin’s group used gold nanoprobes to substitute the conventional fluorescence-labeled probes usually used to report target hybridization [57]. In this approach, the scattered light incident on the gold NPs reported complementary target hybridization. Moreover, using an additional step of silver(I) reduction over the gold nanoprobes surface, the scattering intensity increased and up to 200 fM of genomic DNA target was successfully detected [58]. The detection of single base mismatches was also possible at controlled temperatures with increased selectivity when compared to standard fluorescent probes. Zhou *et al.* added a biomineralization-assisted amplification methodology to this approach that allowed to discriminate single-base mismatches upon a salt-based stringency wash using a target concentration as low as 50 aM [59]. The assay involves two types of derivatized gold NPs: one simultaneously functionalized with both target-recognition oligonucleotide and biomineralization-capable silicatein (probe A) and the other derivatized with an oligo(ethylene glycol) derivative (probe B). These two probes allowed detection of DNA through a cascade signal amplification process: first, a transparent solid support surface modified with a capture oligonucleotide was used to hybridize to both target and probe A in a sandwich manner; then, the silicatein from probe A catalyzes the synthesis of silica simultaneously allowing for the entrapment of probe B and yielding the first signal amplification. The signal can be further enhanced by selective deposition of silver metals on both probes.

Mirkin’s group also developed the microarray approach to detect protein cancer markers using antibody microarrays and gold NPs functionalized with antibodies [60]. This time, they used an electroless gold deposition as a light scattering signal enhancer allowing to detect as low as 300 aM (9,000 copies) of prostate specific antigen in buffer and 3 fM in 10% serum. They have also developed a bio-barcode assay high sensitivity for nucleic acid or protein targets, by combining such microarray approaches with magnetic microparticles holding recognition elements (antibodies or DNA) for specific targets and gold nanoprobes that harbored a secondary recognition agent and hundreds of ssDNA barcodes strands [61,62]. Through this approach they have been able to successfully detected prostate-specific antigen (PSA) in clinical samples with 300 times more sensitivity than commercial PSA immunoassays [63]. In fact, the microarray approach developed by Mirkin’s group is already being commercialized by Nanosphere, Inc. (known as Verigene® system) and was one of the first nanotechnology-based diagnostic platforms to be approved by U.S. Food and Drug Administration (FDA).

Gold NPs functionalized with an antibody anti-CA15-3-HRP were also implemented in a traditional ELISA immunoassay to detect a breast cancer biomarker present in blood (*i.e.*, CA15-3 antigen) such as to improve the optical signal of the assay [64]. When compared to classical ELISA procedures, the gold NPs based ELISA presented a higher sensitivity and shorter assay time allowing to detect a target within the 0–60 U/mL range. Zhang *et al.* also developed a human serum albumin (HSA) immunosensor based on a chitosan-modified glass slide harboring a polymer with amino groups that served as linking sites (using glutaraldehyde) for anti-HAS antibody [65]. The antigen (HSA) and gold NP-labeled anti-HSA were then added to complete the sandwich-type immunocomplex and a linear correlation was obtained between the logarithm of HSA concentration and the absorbance intensity, as measured by UV-visible spectroscopy. Mayer *et al.* have also explored the shifts in the LSPR spectral extinction peak of gold nanorods and bipyramids NPs conjugated with antibodies to monitor real-time dynamic interactions with a specific secondary antibody [66,67].

### 2.3. Infrared Imaging and Enhanced-Spectroscopy

Noble metal NPs presenting a significant absorbance and scattering in the near infrared (NIR) region, such as gold nanoshells, nanorods or silver NPs, have found wide application as contrast agents for *in vivo* imaging [68–70] and also as nanostructures for surface-enhanced infrared absorption spectroscopy (SEIRAS)-mediated biosensing [71].

Several authors have taken advantage of the high permeability of human skin and tissue to NIR radiation to develop minimally invasive deep tissue diagnostic imaging techniques using such NPs as contrast agents. In general, the use of such noble metal NPs in *in vivo* imaging applications allows one to overcome several limitations of conventional NIR organic dyes, such as rapid photobleaching, detection sensitivity, insufficient stability in biological systems and weak multiplexing capability [69]. The high scattering properties of these NPs permits to enhance the contrast of imaging systems based on microscopy, such as dark-field or dual-photon luminescence microscopy, and even combine multiple imaging modalities such as to explore synergistic advantages over single imaging techniques [72,73]. Additionally, the synergy of such biosensing aptitudes with their intrinsic therapeutical capabilities, *i.e.*, theranostics, can provide for more efficient and focused therapies of different diseases, such as cancer [4]. These noble NPs, with a LSPR located within the NIR region, have been applied to different imaging techniques, such as Photoacoustic Imaging (PAI), Photoacoustic Tomography (PAT), dark-field microscopy and two-photon microscopy. PAI and PAT techniques allow noninvasive imaging with the capacity of resolving the optical absorption map of tissue at penetration depths akin with ultrasound imaging. For example, Agarwal and co-workers combined antibody functionalized gold nanorods tuned to the NIR region with PAI to effectively target and image early stage prostate cancer cells. These gold nanorods allowed them to produce high contrast images between targeted tissue and non-targeted tissue [74]. Similarly, Eghtedari *et al.* used gold nanorods as a contrast agent for *in vivo* detection using a laser based PAI system [75], while Pan *et al.* used NIR absorbing 2–4 nm gold nanobeacons (*i.e.*, gold NP functionalized with ssDNA strands that form a hairpin structure) to detect sentinel lymph nodes through PAI in *ex vivo* tissue samples [76]. On the other hand, Wang’s group has used PAT technique to image the distribution of gold nanoshells circulating in the vasculature of a rat brain by achieving a gradual enhancement of the NIR optical absorption in the brain vessels [77,78]. They concluded that these NPs presented a high spatial resolution and enhanced sensitivity with an increased photoacoustic contrast up to 81% greater than blood, in their latest report. Lu *et al.* have also observed this increase in contrast by using similar NIR absorbing 40 nm gold nanoshells functionalized with thiolated-PEG as contrast agents for PAT in nude mice [79].

NIR scattering gold nanoshells were used by Loo and co-workers as a contrast agent in dark-field microscopy to target human epidermal growth factor receptor 2 (HER2), a clinically significant breast cancer molecular marker [80]. Similarly, Bickford *et al.* also used these type of gold nanoshells to image live HER2-overexpressing cancer cells using two-photon microscopy [81].

The LSPR of noble metal NPs when tuned into the NIR part of the spectrum can also be exploited for surface enhanced infrared absorption (SEIRA) spectroscopy, taking advantage of the direct NIR excitation of molecules adsorbed to metal NPs’ surface. Some applications have benefited from SEIRA, namely in chemical analysis and biochemical sensing [82]. Mostly, gold and silver NPs have been used for the development of SEIRA based biosensors. As an example, by depositing gold NPs functionalized with specific antibodies over a SiO_2_/Si wafer surface, Enders and co-workers were able to detect antibody-antigen coupling with a 25-fold enhancement over a SiO_2_/Si wafer surfaces’ lacking the gold NP layer [71]. The IR transmission spectra (normal incidence) of non-specific antibody–antigen coupling and of specific antibody–antigen coupling was studied using a monolayer of anti-rabbit antibodies from goat immobilized on gold NP/(aminopropyl)-triethoxysilane/SiO2/Si wafer as reference. The specific antibody–antigen reaction could clearly be detected via the characteristic absorption bands of proteins that are not coupled to the non-specific antigens.

### 2.4. Raman-Spectroscopy and Imaging

In 1974, Fleishmann and co-workers demonstrated for the first time that Raman signals from different types of molecules could be dramatically enhanced by their adsorption to silver surfaces [83], as well as on other metals, as later demonstrated. Since then, the use of metal NPs for surface-enhanced Raman scattering (SERS), mostly gold and silver NPs, has led to the development of a wide variety of new biosensors for the detection of nucleic acids, antibodies, proteins and other biological molecules [84,85]. Raman scattering arises from the inelastic scattering of photons that hit the analyte molecule and either gain or lose energy from the molecule’s vibrational and rotation motion. This interaction generates a very narrow spectrum of bands that is unique for each analyte, and which can be greatly enhanced by metal nanostructures in the order of 10^5^ to 10^6^ times more than non-SERS Raman signals [86]. This enhancement is mainly attributed to the LSPR of the metal nanostructures and can be greater enhanced when the analyte molecules are placed in “hot spots” where LSPR fields overlap, such as junctions between aggregates of noble metal NPs [87].

The SERS detection of specific biomolecules mediated by noble metal NPs can be either accomplished directly or indirectly, through the association with a molecule with an intense and characteristic Raman signature, typically a fluorescent dye. Most of the methods based on SERS have taken similar approaches to those already described in colorimetric and fluorescent assays. For example, by exploring the electrostatic adsorption of some biomolecules to the NPs’ metal surface some groups have created a metallic layer made of gold or silver NP aggregates to perform the detection of biomolecules and metabolites by SERS. Gogotsi *et al.* developed a SERS label-free biosensor based on a glass coated with gold NPs to detect and quantify nicotinic acid adenine dinucleotide phosphate (NAADP) molecules, which is a calcium secondary messenger that plays a crucial role for intracellular Ca^2+^ release [88]. This system allowed for the rapid detection of 100 μM NAADP without any special sample purification or labeling, making it an important tool for the study of normal cell function and cancer development. Ozaky’s group used a similar approach using a surface covered with silver NPs instead of gold to detect bombesin, a neurotransmitter tumor marker, and its modified analogues by SERS [89]. They have also developed a protocol to monitor protein-protein and protein-small molecule interactions employing colloidal silver staining of the samples for producing active substrates for SERS [90,91]. Similarly, Van Duyne’s group used SERS to detect and monitor glucose levels by adding a self-assembled monolayer of decanethiol over an aggregated silver NPs film in order to increase glucose adsorption which is minimal for bare-SERS active substrates, such as roughened silver [92,93]. The initial glucose detection limit of this system was lower than 5 mM, but was subsequently improved by the use of mixed decanethiol and mercaptohexanol or (1-mercaptoundeca-11-yl)tri(ethylene glycol) layers and use of gold NPs instead of silver [94–96].The detection of ssDNA using SERS was carried out by Fabris and co-workers using a slide derivatized with uncharged peptide nucleic acids (PNAs) that recognized specific negatively charged ssDNA targets, which upon hybridization mediated the adsorption of positively charged silver NP [97]. A further addition of rhodamine6G dye to this system gave rise to SERS signals that allowed monitoring the hybridization event.

**Figure 2.**
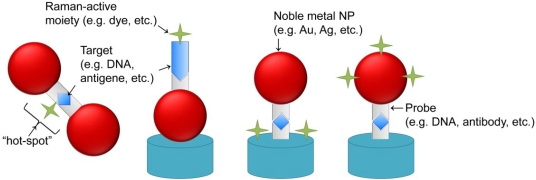
Examples of different variations of cross-linking approaches for SERS. The schematics depicts different approaches to the use of Raman-active moieties to enhance SERS signal output via cross-linking strategies: the Raman-active moiety can be bond to the target (two illustrations to the left) or to the surface of the film and/or NP (two illustrations to the right). Signal enhancement will occur upon cross-linking of the involved moieties derived from target recognition and binding.

The use of conjugated noble metal NPs was also explored in SERS-based biosensors for a more versatile and specific detection of biomolecules. In most cases, a probe (e.g., DNA, antibody) is functionalized to the NP or an aggregated layer of NPs such as to create a sandwich conjugation with a Raman-labeled probe upon target detection, thus generating the SERS signal. Some variations of this cross-linking approach (Figure 2) have been explored by (i) Bonham *et al.* in the detection of DNA-binding proteins through the use of DNA functionalized gold NPs and an electroless silver plating of the conjugates to enhance the output signal [98]; (ii) Ryu *et al.*, to detect a specific Anthrax biomarker by means of peptide derivatized gold NPs [99] (iii) Vo-Dinh and co-workers for the detection of specific DNA sequences, such as HIV DNA, using Raman-active dye-labeled DNA on silver or gold NPs [100–103]; (iv) Ozaki’s group to detect hepatitis B virus antigen using surface immobilized antibodies and gold NPs conjugated to antibodies and 4-mercaptobenzoic acid (MBA), acting as a Raman-active probe. In this case the signal was further enhanced by silver staining allowing to detect as low as 0.5 μg/mL of target [104]; (v) Driskell *et al.* in the detection of viral pathogens, such as feline calicivirus, using gold NP conjugated with a monoclonal antibody and a Raman reporter moiety, namely 5,5′-dithiobis(succinimidyl-2-nitrobenzoate) [105]; among others [106–108].

The cross-linking approach can also be used to entrap the Raman reporter moiety in “hot-spots” between NPs upon target detection, benefiting from an additional enhancement of SERS signal. For example, ssDNA functionalized silver [109] or gold [110,111] Raman dye-labeled NPs were used to detect the presence of a complementary ssDNA target that would generate a cross-linked nanostructure of NPs and, therefore, entrap the Raman-dye within it.

Vo-Dinh’s group also explored a molecular nanobeacon approach using a Raman dye-labeled DNA functionalized on silver NPs where the presence of a complementary target would decrease SERS signal. This decrease was a consequence of the disruption of the hairpin structure that, otherwise, leads the Raman dye to the proximity of NPs’ metallic surface [112]. Through this approach they were able to detect and quantify a gag gene sequence of the human immunodeficiency virus type 1 (HIV-1) within the range of 0.5 and 2 μM of DNA target. They have also expanded this method for multiplexing capability using two different Raman dyes-labeled gold nanobeacons to successfully detect the presence of the erbB-2 and ki-67 breast cancer biomarkers [113].

Other multiplex SERS methods have also been developed for biosensing based on a microarray approach [114,115], multiple dye-labeling [116,117], or even on “bar-coded” noble metal NPs [118–120]. An example of the latter, are the gold barcode nanodisks fabricated by Mirkin’s group using on-wire lithography to create a barcode with 2 nm separated patterns of nanodisks over nickel wires [119]. The gaps located between the gold nanodisks acted both as “hot-spots”, to enhance the SERS signal from the Raman-active dyes, and as a physical encoded “barcode” pattern, for multiplex detection of biomolecules using confocal Raman spectroscopy. As proof-of-concept, they demonstrated that multiplexing detection of DNA was possible with targets concentrations as low as 100 fM. More recently, the group also developed silver nanorods with etched gaps for multiplex biosensing by SERS [120].

The application of SERS mediated by noble metal NPs has also been described for *in situ* detection of specific reactions and intracellular molecular imaging of living cells and tissues. As an example, Qian and co-workers derivatized 60–80 nm gold NPs with PEG and a tumor targeting ligand, namely single-chain variable fragment antibodies, to detect tumors both *in vitro* and *in vivo* through SERS imaging [121]. These NPs were able to detect tumor biomarkers, such as epidermal growth factor receptors on human cancer cells and in xenograft tumor models, being more than 200 times brighter than NIR-emitting quantum dots (QDs). They were able to detect tumors up to 2 cm below the skin of live animals, mainly due to the fact that the LSPR peaks of such NPs was tuned within the 630–785 nm region, where the optical absorption of water is minimal. Specific cancer markers (phopholipase Cg1 biomarker proteins—PCg1) were also imaged in live human embryonic kidney cells (HEK293) by SERS microscopy using derivatized gold/silver core/shell NPs with R6G Raman tags and IgG antibodies [122]. Multiplexed SERS imaging has also been demonstrated in living mouse upon the injection of five different 60 nm gold NPs derivatized with a silica shell and a unique Raman active moiety for each NP (making up a total of 120 nm NP in diameter) [123]. These conjugated SERS-NPs naturally accumulated in the liver 24 h after the injection and presented five distinguishable SERS spectra signals up to 5 mm of tissue depth, which limited the mapping of the total liver for deeper tissue levels.

### 2.5. Fluorescence Spectroscopy

**Figure 3.**
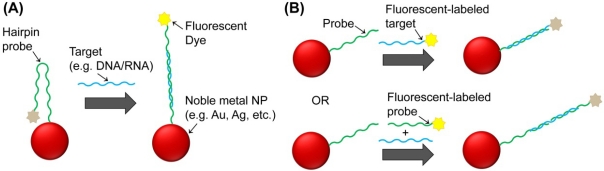
Different approaches for fluorescent-based noble metal NPs biosensing. (**A**) molecular nanobeacons and (**B**) other nanoprobes. Distance to the nanoparticle determine the fluorescence signal that is observed. Distances are not represented to the scale.

The exceptional quenching of fluorescent dyes mediated by noble metal NPs has allowed increased sensitivity and efficiency of Förster resonance energy transfer (FRET)-based biosensors. Two approaches have been used thus far: the so called molecular beacons that rely on NPs functionalized with fluorescent-labeled ssDNA strands that form a hairpin structure; and noble metal nanoprobes consisting of NPs functionalized with single strand nucleic acid strands that are hybridized to another fluorescent-labeled single strand nucleic acid probe (Figure 3).

In the case of the molecular beacons, the hairpin structure leads the fluorescent dye moiety to the proximity of the NP’s surface and, consequently, to the quenching of fluorescence due to Nanoparticle Surface Energy Transfer (NSET) occurring between the dye (donor molecule) and the NP’s surface (acceptor)—a phenomenon that has been described to permit energy transfer for distances nearly twice as far as FRET [124,125]. In the presence of a complementary DNA/RNA target, the hairpin structure is disrupted by target hybridization and fluorescence is restored. Several methods have been developed based on this approach to monitor specific nucleic acid hybridizations as well as cleavage processes mediated by nucleases, mainly using gold NPs [126]. For example, Dubertret and co-workers reported the use of molecular nanobeacons based on 1.4 nm gold NPs to detect single-base mismatches in DNA with a 100-fold increase in sensitivity when compared to conventional molecular beacons [127]. Similarly, Benia *et al.* used molecular nanobeacons based on 13 nm gold NPs to successfully detect a mutation associated to cystic fibrosis using just 1 nM of target [128]. They have also shown that the use of gold NPs larger than 13 nm for molecular nanobeacons is not recommended due to the fact that larger NPs present relevant quenching efficiency even at large fluorophore–NP separation lengths, despite the fact that they could carry a higher number of molecular beacons.

On another approach, noble metal nanoprobes can be combined with dye-labeled ssDNA probes to detect specific nucleic acid targets by FRET/NSET mediated mechanisms. One method is to design such probes to harbor complementary and contiguous sequences to the target, in such way that upon target hybridization the dye is forced to approach the NP’s surface and the fluorescent signal consequently decreases [129]. Another method is to hybridize the dye-labeled ssDNA directly to the nanoprobes and detect specific target DNA sequences based on strand displacement of the fluorescent probe [130]. For example, in the presence of a fully complementary long ssDNA targets, the short dye-labeled DNA strand is displaced and, as a result, the fluorescence that was initially quenched by the NP is restored. Through this approach, the signal-to-noise ratio and detection limit of 50 pM is significantly improved when compared with similar probes using organic acceptors as reported by Mo and co-workers [130]. A similar approach was used by Wang and co-workers to detect proteins using the dye-labeled ssDNA hybridized to an aptamer that is immobilized on gold NPs [131]. Whenever a protein binds to the aptamer, the dye-labeled ssDNA is released and fluorescence is restored, allowing to detect proteins with high sensitivity and specificity. Similarly, Ray and co-workers demonstrated that the DNA cleavage by specific nucleases can also be easily and quickly monitored using Au-nanoprobes hybridized with complementary Cy3-labeled nucleic acid strands with a sensitivity several orders of magnitude greater than the conventional gel electrophoresis or HPLC techniques, or even a few orders of magnitude greater than UV assays [132]. The presence of a S1 nuclease leads to the cleavage of the dye-labeled dsDNA bound to the NP and consequently to an enhancement of the fluorescence signal by a factor of 120. Jennings *et al.* also developed a method based on this approach using NSET to measure Mg^2+^-induced conformational changes and kinetics of a hammerhead ribozyme using a fluorescent labeled RNA that hybridized with a complementary strand attached to 1.4 nm gold NPs and formed the hammerhead structure [133]. Mayilo *et al.* described a sandwich immunoassay using gold NPs quenching upon detecting a cardiac troponin T protein by its interaction with two different antibodies, one attached to gold NPs and the other labeled with fluorescent dyes [134]. Guirgis and co-workers developed an immunoassay to detect malaria based on the fluorescence quenching of Cy3B-labeled recombinant *Plasmodium falciparum* heat shock protein 70 (PfHsp70) upon binding to gold NPs functionalized with an anti-Hsp70 monoclonal antibody [135]. Upon competition with the free antigen, the Cy3B-labeled recombinant PfHsp70 is released to solution resulting in an increase of fluorescence intensity. The interaction of human blood proteins with gold NPs has also been studied by letting the proteins interact with fluorescent gold NPs and performing the detection through quenching [136].

The combination of noble metal NPs and semiconductor QDs as a FRET donor–acceptor couple has also been explored by several authors to develop fluorescence competition assays for nucleic acid, protein and antibody/antigen detection following the same approaches described above, simply by substituting the dye with QDs [137–139]. Additionally, more than one dye can also be simultaneously used to develop multiplexing approaches of all the previously described methods by tracking the specific emission of each dye, since the quenching effectiveness of noble metal NPs is widespread across the UV/visible spectra. Namely multiplex methods for DNA detection [140,141], endonucleases monitoring [142], small analytes detection (adenosine, potassium and cocaine) using aptamers [143] have been described.

The use of noble metal NPs to enhance the fluorescence signal of dyes used in a DNA assay has been reported by Dragan *et al.* [144]. A 2-color DNA assay based on the combination of the Metal-Enhanced Fluorescence (MEF) effect and microwave-accelerated DNA hybridization allowed to detect dye-labeled targets in a microarray format. The microarray ssDNA probes are functionalized over a monolayer film of silver NPs which contributed for the large enhancement of the target’s fluorescent label due to a short-range (0–30 nm) coupling effect of a dye’s excited state electronic system with NPs plasmons.

Different noble metal NPs with inherent fluorescent properties or directly functionalized with fluorescent dyes have also been used for *in vivo* biosensing. Zhang and co-workers have developed fluorescent metal nanoshells composed of silica spheres with encapsulated [Ru(bpy)3]^2+^ complexes as cores and thin silver layers as shells to detect single microRNA molecules in cells positive to lung cancer [145]. These nanoshells harbored a covalently bound ssDNA probe that recognized the micro RNA (miRNA) target in the cells and presented highly contrast images against cellular autofluorescence, mainly due to their strong intensity emission and longer lifetimes. The same authors have also reported similar fluorescence enhancements of different fluorophores when functionalized to different silver NPs, which were used to target different receptors within cell membranes [146,147]. Using a different approach, Phillips and co-workers developed an array of gold NP–conjugated polymer constructs for bacterial sensing [148]. The efficient quenching ability of gold NPs coupled with the molecular-wire effect of conjugated polymer compounds generated a pronounced fluorescence response mediated by the binding strength of the bacterium to the gold NP. Through this approach, the authors successfully differentiated twelve different bacteria using only three systems.

### 2.6. Electric and Electrochemical Approaches

The unique physicochemical properties of noble metal NPs, such as high surface area, high mechanical strength, rich electronic and catalytic properties, have been explored to detect biological recognition events through electrical signals or electrochemical transductions and design a new generation of electronic biosensor devices [149,150]. These electrical and electrochemical approaches generally rely on the changes in the ohmic response of an electrical circuit or the flow of electrons arising from faradaic processes (*i.e.*, oxidation or reduction) near the surface of an electrode, respectively, to achieve biosensing. Although the direct electrical and electrochemical detection of biomolecules is possible, many have benefited from the electroactive or catalytic properties of NPs as reporters for biosensing with unprecedented levels of sensitivity [151].

The key aspect of electrical biosensors is the generation or modulation of electrical current in an electronic circuit by molecular binding of the biomolecule of interest, such as the completion of a broken circuit or altering the electrical properties of an electro-mechanical sensor (e.g., piezoelectric sensor). An example of the latter case, is the piezoelectric biosensor developed by Chen and co-workers using gold NP functionalized with DNA probes for the detection of the foodborne pathogen *Escherichia coli* O157:H7 [152]. In this application, the authors used gold nanoprobes as a mass enhancer and, thus, further amplify the changes in the signal and the sensitivity of the piezoelectric sensor, as well as increase the specificity of the method. The oscillation frequency of the piezoelectric sensor decreased in real-time as the weight at the sensor's surface increased due to the sandwich hybridization occurring between the target oligonucleotide, the sensor’s probe oligonucleotides and the circulating DNA functionalized gold NP. Similarly, Pang *et al.* used this piezoelectric approach to detect a DNA single-base mutation within an artificial codon CD17 of the β-thalassemia gene, complementing the method with a DNA ligase reaction [153]. Immunoassays were also developed using piezoelectric sensors in which the specific agglutination of antigen-coated gold NPs causes a frequency change in the signal when in the presence of the corresponding antibody [154,155].

Another electrical approach for biosensing is to explore the conductivity of noble metal NPs and use them as conductive tags to act as a “switch” on an electrical circuit. Following this idea, several groups developed DNA sensors based on electrode gaps in which the binding of noble metal NPs functionalized with oligonucleotides leaded to conductivity changes associated with target-probe binding events [156–160]. In the case of the Mirkin’s group approach [156], the presence of a complementary target allowed the gold nanoprobes to form a sandwich hybridization with the probes functionalized within the electrode gaps, thus filling the gaps and facilitating silver deposition to further increase conductivity between electrodes, allowing to detect DNA concentrations as low as 500 fM with a single base resolution.

In general, electrochemical biosensors employ potentiometric, amperometric or impedimetric transducers to convert the biosensing information into a measurable signal. Some authors have described the use of potentiometric stripping analysis to detect and quantify gold, silver or gold/silver NPs that worked as tags for biosensing DNA [161–164], antibodies [165–167] or aptamers [168], with sensitivities of down to 600 aM, 3 fM and 1 nM, respectively. Others have described the use of cyclic voltammetry [169,170], differential pulse voltammetry [171], chronocoulometry [172] electrochemical methods to perform biosensing of DNA and proteins mediated by noble metal NPs acting as electrochemical labels. Ozsoz and co-workers described an electrochemical genosensor based on gold NP and disposable pencil graphite electrodes for the detection of a Factor V Leiden mutation in PCR products amplified from clinical samples [173]. The method allowed detection of as little as 0.78 fmol of PCR amplicons using the oxidation signal of colloidal gold nanoprobes that hybridized to the complementary DNA targets. More recently, Yu *et al.* used gold NP in combination with apoferrition NPs to further increase the sensitivity of electrochemical DNA biosensing down to 51 aM [174].

Several authors have described the development of amperometric-based biosensors, which are usually more suited for mass production than potentiometric biosensors [175]. In this approach the working electrode is usually a noble metal NP covered by the biorecognition component, which enables the amperometric signal. Namely, using this amperometric approach: Polsky and co-workers used platinum NPs functionalized with nucleic acid aptamers to detect thrombin with a sensitivity limit corresponding to 1 nM [176]; Munge *et al.* developed an immunosensor to detect attomoles of the cancer biomarker interleukin-8 using glutathione derivatized gold NPs [177]; Jensen and co-workers used inkjet printed arrays with gold NPs to detect the cancer biomarker interleukin-6 directly in serum with a detection limit of 20 pg/mL [178]; Yin *et al.* used gold NPs over a surface of poly(styrene-acrylic acid) nanospheres, that served as a matrix to conjugate alkaline phosphatase, to detect the tumor necrosis factor α up to 0.01 ng/mL [179]; Meng *et al.* used gold NPs combined with ferric oxide NPs and carbon nanotubes to detect up to 0.04 ng/mL of the tumor biomarker alpha fetoprotein [180]; Sharma and co-workers used gold NPs and screen printed electrodes modified with multiwall carbon nanotubes to detect *Plasmodium falciparum* histidine-rich protein 2 in serum of humans with malaria [181]. In this case, the authors compared the method with a commercial kit, Paracheck Pf test (Orchid Biomedical Systems, Goa, India), and determined that their method was more sensitive and specific than the kit, having a detection limit of 8 ng/mL; Merkoçi’s group used gold NP and magnetic beads to develop a magneto-immunosensor for the detection of anti-hepatitis B virus antibodies and human IgG in human serum [182,183]; Che and co-workers used gold NPs with multi-walled carbon nanotube-silver composites to detect down to 0.08 ng/mL of alpha-1-fetoprotein [184]; Scodeller *et al.* used gold-silver core-shell NPs to develop a system responsive to glucose based on the enzymatic reduction of FAD by glucose [185].

Some impedimetric based electrochemical biosensors have also been reported, following either impedance (Z) or its components resistance (R), capacitance (C) and inductance (L). Mahmoud and co-workers developed an assay based on a modified gold electrode with single-walled carbon nanotube/gold NPs derivatized with thiol terminated ferrocene-pepstatin conjugates able to detect picomolar levels of HIV-1 protease and screen its potent inhibitors using electrochemical impedance spectroscopy [186]. Yang *et al.* fabricated a capacitive immunosensor based on grafted ethylene diamine and self-assembled gold NPs monolayer on glassy carbon electrode for the detection of *Salmonella* spp. using also electrochemical impedance spectroscopy [187]. This assay was successfully used for the detection of *Salmonella* spp. in lab-processed commercial pork samples and presented a detection limit of 100 CFU/mL.

Recently, Tian and co-workers developed an electrochemiluminescence biosensor using luminol functionalized gold NPs as labels [188,189]. The electrochemiluminescence of luminol is enhanced 2 to 3-orders of magnitude on gold NPs when compared to bare gold electrodes and allowed this sandwich-type immunocomplex sensor to achieve a detection limit of 1 pg/mL for human IgG in human sera.

Multiplex approaches based on some of the methods previously described have also been described. Li and co-workers used gold surface DNA arrays functionalized with multiple probes in combination with reporting probes made of silver NPs to detect herpes simplex virus (HSV), Epstein-Barr virus (EBV) and cytomegalovirus (CMV) sequences by differential pulse voltammetry [190]. The silver NPs-based tag allowed for the detection down to 5 aM of target DNA by producing a 103-fold amplification in the differential pulse voltammetry signal, due to the excellent electroactive properties of silver NPs.

Enzyme-functionalized gold NPs harboring specific DNA sequences were also used as catalytic labels for multiplex DNA detection [191]. A sandwich hybridization strategy was used in this method, involving DNA probes functionalized on a glassy carbon electrode which registered the electrochemical signals generated by the enzymatic products produced from the substrates catalyzed by the functionalized enzymes, namely horseradish peroxidase (HRP) and alkaline phosphatase (ALP), upon complementary target recognition. This method allowed to detect two distinct targets in a one-pot solution with a detection limit of 0.1 and 12 fM using HRP–gold NPs and ALP–gold NPs as labels, respectively. Zhang *et al.* explored anodic stripping voltammetry using a screen-printed carbon electrode chip in combination with bio-barcoded gold NPs, as electrochemical reporters, and magnetic NPs, for easy and clean separation from the sample, to detect multiple pathogens (e.g., *Bacillus anthracis* and *Salmonella enteritidis*) [192]. A multiplex immunoassay was also developed using screen-printed array of carbon electrodes and gold NPs as electrochemical labels [193]. This immunoassay is performed using a sandwich approach between the electrode array probes and gold NPs labeled with antibodies, mediated by the target analytes (*i.e.*, human and goat IgG). Under optimal conditions the method allowed to detect a minimum target concentration of 1.1 and 1.6 ng/mL, respectively, using differential pulse voltammetry.

## 3. Application in Biosensing Platforms—Future Perspectives

Noble metal NP-based biosensors provide a new horizon for novel functions with a variety of applications in clinical diagnostics and biological research. This review has provided a summary of the various biosensing strategies developed thus far, that explored the unique physicochemical properties of noble metal NPs, towards their potential applications in clinical and point-of-care diagnostics. Table 2 summarizes the different types of real biological samples that have been tested with these noble metal NP-based biosensors, according to their methodology principle.

Noble metal NPs have already proven to be one of the most important groups of nanomaterials for biosensing approaches, as well as in other biomedical applications (e.g., therapy). Highly sensitive and specific biosensors based on noble metal NPs have open up the possibility of creating new diagnostic platforms for disease markers, biological and infectious agents in the early-stage detection of disease and threats. Among the different approaches explored thus far, such as colorimetric, NIR imaging, fluorescence quenching/enhancement, SERS, electric and electrochemical sensing, there are, as always, advantages and disadvantages associated to each approach towards the ultimate diagnostic application in real samples. In most cases, the proof-of-concept for such approaches has been reported using controlled synthetic bioanalytes and/or samples, while validation of these biosensors with a statistically larger population of real samples still remains to be addressed as to further prove the potential application in clinical and point-of-care diagnostics. Apart from that, one key issue to take into consideration in biosensing platform integration is the increasing demand for higher sensitivity and selectivity at minimal costs, and the possibility to monitor biosensing in real-time, especially for point-of-care platforms, for which simplicity should also be considered. Considering this, colorimetric and electrochemical approaches are the most promising due to their simplicity, high sensitivity and specificity, in addition to the fact that in most cases they simply do not require expensive and complex instrumentations to achieve a sample-in/answer-out biosensing platform. Moreover, such approaches allow for multiplexing biosensing enabling their application in a range from low- to high-throughput diagnostics. In fact, some of these approaches have already reached commercialization, as is the case of Verigene^®^ system by Nanosphere, Inc. (Northbrook, IL, USA) which explores the scattering capabilities of gold NPs in a microarray platform providing a diagnostic platform for DNA/RNA and protein sensing.

On the other hand, the use of NIR active noble metal NPs as contrast agents for PAI or PAT is one of the most promising platforms for *in vivo* imaging, provided that aspects concerning the toxicology of such nanomaterials are carefully addressed. Moreover, in most cases, the use of such NP in *in vivo* imaging allows also to simultaneously carry out therapeutic procedures (*i.e.*, theranostics), such as photothermal therapy, enabling a more precise and efficient treatment of diseases, such as cancer. As an example of this potential, Nanospectra Biosciences (Houston, TX, USA) is already carrying out clinical trials for its AuroLase^®^ Therapy approach, which combines gold nanoshells with photothermal ablation technology for *in vivo* imaging and therapy.

Other developed methodologies using noble metal NPs, such as SERS and fluorescent quenching/enhancement approaches, can still find great application in research laboratories, allowing to unveil current paradigms and emerging challenges of biology and medicine. That said, some of the described noble metal NP-based biosensors that are currently under development will most likely revolutionize our understanding of biological mechanisms and push forward the clinical practice through their integration in future diagnostics platforms. As nanotechnology is becoming increasingly accessible to research laboratories and, most recently, to clinical laboratories, significant advances in the area of noble metal NP-based biosensors will certainly occur in the near future, pushed by the intervention of a cross-disciplinary team, consisting of a variety of researchers with diverse backgrounds, that will make the prospect of widespread use of noble metal NPs in clinical and point-of-care diagnostics a reality.

## Figures and Tables

**Table 1.** Types of conjugations between biomolecules and noble metal NPs.

**Type of conjugation**	**Pros**	**Cons**
**Electrostatic interactions** (e.g., adsorption of negative charged DNA to positive charged gold NP)	- Very simple and straightforward to perform.	- Restricted to opposite charged biomolecules and NPs; - Very sensitive to environmental properties (e.g., pH, ionic strength, *etc*.); - Weak functionalization.
**Chemisorption** (e.g., quasi-covalent binding of thiol-functionalized biomolecule to gold NP)	- Allows oriented functionalization; - Very robust functionalization.	- Requires NPs with capping agents with weaker adsorption than the derivatization moiety; - Usually requires modification of the biomolecule; - Subject to interference by other chemical groups available for adsorption within the biomolecule; - Affected by chemical degradation and surface oxidation of some NPs (e.g., silver).
**Affinity-based** (e.g., His-tag protein binding to Ni-NTA derivatized gold NP)	- Allows oriented functionalization; - Very straightforward binding between affinity pairs.	- Requires modification of both NPs and biomolecules with an affinity pair; - Limited to availability of suitable binding affinity pairs.

**Table 2.** Summary of real biological samples tested with noble metal NP-based biosensors according to the type of NP and explored methodology principle.

**Method**	**Type of NP**	**Target/Samples [reference]**
Colorimetric/scanometric	Gold	SNP (rs2131877) in human DNA [32] Mutations in EGFR gene in genomic DNA [33] SNP associated with long QT syndrome in genomic DNA [31] SNPs in β-thalassemia gene in genomic DNA—mediated by PCR [43,45] SNPs in MBL2 gene in genomic DNA—mediated by PCR [53,54] Fusion genes in K562 cell line [55] Hepatitis C virus RNA [35] *M. tuberculosis* DNA and associated drug-resistance SNPs in clinical samples [46,48,50] BCR-ABL fusion transcript in clinical samples [47] FSY1 mRNA in total RNA [49]
	Gold	Melamine in whole milk [40] Prostate specific antigen (PSA) in human serum [63] CA15-3 breast cancer biomarker in human serum [64] Genetically modified organisms [52] Mutations associated to methicillin resistance in S. aureus & Factor V Leiden mutants [58]
	Gold/silver alloy	TP53 gene [28] BCR-ABL fusion genes [29]
NIR	Gold	Prostate cancer cells in mouse [74] Lymph nodes in mouse [76] Brain vessels in mouse [77–79] HER2 cancer biomarker in breast adenocarcinoma cells [80,81]
SERS	Gold	Nicotinic acid adenine dinucleotide phosphate (NAADP) in cell extracts [88] Glucose in rat [96] Multiple pathogen DNA in clinical specimens (cerebrospinal fluid, stool, pus, and sputum) [118] Feline calicivirus (FCV) antibody from cell culture media [105] Prostate-specific antigen in human serum [194] Deep-tissue imaging in living mouse [123]
	Silver	HIV-1 DNA in genomic DNA - PCR mediated [112] Glucose in rat [93]
	Gold/silver core/shell	Phospholipase Cγ1 biomarker protein in cancer cells [122]
Fluorescence-based	Gold	*Plasmodium falciparum* heat shock protein in infected blood cultures [135]
	Silver	miRNA-486 expression levels in lung cancer cells [145] Cell membrane imaging in cell lines [146,147]
Electric/Electrochemical	Gold	Factor V Leiden DNA mutation in genomic DNA – mediated by PCR [173] *E. coli* O157:H7 in food samples [152]
	Gold	Anti-*Toxoplasma gondii* immunoglobulins in rabbit blood and serum [154] Human and mouse IgG antibody in human and mouse serum [166] Interleukin-8 (IL-8) cancer biomarker in human serum [177] Interleukin-6 (IL-6) cancer biomarker in calf serum [178] Tumor necrosis factor α (TNF-α) in human serum [179] *Plasmodium falciparum* histidine-rich protein 2 in serum [181] Anti-hepatitis B virus antibodies and human IgG in human serum [182,183] HIV-1 protease and inhibitors in human serum [186] *Salmonella* spp. in pork samples [187] Human IgG in human serum [188,189] Human and goat IgG in serum [193]
	Silver	α-1-Fetoprotein (AFP) in human serum [184]
